# Biomimetic 3D Environment Based on Microgels as a Model for the Generation of Drug Resistance in Multiple Myeloma

**DOI:** 10.3390/ma14237121

**Published:** 2021-11-23

**Authors:** Juan Carlos Marín-Payá, Blanca Díaz-Benito, Luis Amaro Martins, Sandra Clara Trujillo, Lourdes Cordón, Senentxu Lanceros-Méndez, Gloria Gallego Ferrer, Amparo Sempere, José Luis Gómez Ribelles

**Affiliations:** 1 Centre for Biomaterials and Tissue Engineering, CBIT, Universitat Politècnica de València, 46022 Valencia, Spain; juancarlosmarinpaya@gmail.com (J.C.M.-P.); bldiaz03@ucm.es (B.D.-B.); luisamaromartins@gmail.com (L.A.M.); sanclatr@gmail.com (S.C.T.); ggallego@ter.upv.es (G.G.F.); 2 Biomedical Research Networking, Center on Bioengineering, Biomaterials and Nanomedicine (CIBER-BBN), 28029 Valencia, Spain; 3 Grupo de Investigación en Hematología, Instituto de Investigación Sanitaria La Fe (IISLAFE), 46026 Valencia, Spain; lou.cordon@gmail.com (L.C.); sempere_amp@gva.es (A.S.); 4 Centro de Investigación Biomédica en Red de Cáncer (CIBERONC), Instituto Carlos III, 28029 Madrid, Spain; 5 BCMaterials, Basque Center for Materials, Applications and Nanostructures, UPV/EHU Science Park, 48940 Leioa, Spain; senentxu.lanceros@bcmaterials.net; 6 IKERBASQUE, Basque Foundation for Science, 48013 Bilbao, Spain; 7 Physics Centre, Campus de Gualtar, University of Minho, 4710-057 Braga, Portugal; 8 Haematology Department, Hospital Universitari i Politècnic La Fe, 46026 Valencia, Spain

**Keywords:** biopolymers, biocomposites, multiple myeloma, microgel, hyaluronic acid, collagen

## Abstract

The development of three-dimensional environments to mimic the in vivo cellular response is a problem in the building of disease models. This study aimed to synthesize and validate three-dimensional support for culturing monoclonal plasma cells (mPCs) as a disease model for multiple myeloma. The three-dimensional environment is a biomimetic microgel formed by alginate microspheres and produced on a microfluidic device whose surface has been functionalized by a layer-by-layer process with components of the bone marrow’s extracellular matrix, which will interact with mPC. As a proof of concept, RPMI 8226 cell line cells were cultured in our 3D culture platform. We proved that hyaluronic acid significantly increased cell proliferation and corroborated its role in inducing resistance to dexamethasone. Despite collagen type I having no effect on proliferation, it generated significant resistance to dexamethasone. Additionally, it was evidenced that both biomolecules were unable to induce resistance to bortezomib. These results validate the functionalized microgels as a 3D culture system that emulates the interaction between tumoral cells and the bone marrow extracellular matrix. This 3D environment could be a valuable culture system to test antitumoral drugs efficiency in multiple myeloma.

## 1. Introduction

Multiple myeloma (MM) is a B-cell hematologic malignancy characterized by a monoclonal plasma cell (mPC) infiltration with heterogeneous localization in the bone marrow compartment [1,2]. The mPCs accumulate in the bone marrow microenvironment through interactions between bone marrow extracellular matrix components and resident cells receiving multiple signals, which promote their survival and drug resistance [3,4,5].

One of the resistance phenomena involved in MM is the cell adhesion mediated drug resistance (CAM-DR) [1,6]. The adhesion of tumoral cells to the components of the extracellular matrix or to the bone marrow stromal cells [1,7,8] confers a multi-drug resistance phenotype [6,7]. The bone marrow extracellular matrix mainly consists of laminin, type I and IV collagen, fibronectin, glycosaminoglycans heparan sulphate, hyaluronic acid, and chondroitin sulphate [1,9]. In MM, hyaluronic acid promotes proliferation in IL-6-dependent myeloma cells [10], as well as drug resistance to dexamethasone (DEX) [11]. In the case of collagen, syndecan-1 mediates the adhesion of tumor cells to it via heparan sulfate chains. Syndecan-1 plays a role in mediating MM cell interactions with the bone marrow niche [12,13], and recently it has been shown that syndecan-1 promotes Wnt/β-catenin signaling in MM. Wnt signaling has been related to proliferation, dissemination, drug resistance, and disease progression [14,15].

Given the influence of the microenvironment on the resistance and development of the disease, it is not surprising that the expansion of primary MM cells outside the bone marrow niche has been unsuccessful [16,17,18]. The survival and growth of primary human MM cells have been demonstrated in co-culture with mesenchymal cells, osteoblasts, osteoclasts, macrophages, and dendritic cells [19,20,21,22]. Besides, it has been evidenced that cell responses in 3D cultures are similar to in vivo behavior, unlike 2D cultures [16,23]; therefore, current research focuses on the design of 3D culture systems to reproduce mPC behaviors and their susceptibility to drugs mediated by the bone marrow microenvironment.

The goal of our proposal was to use a biomimetic microgel to recapitulate the environment of tumor cells in vivo. The culture platform proposed in this work to create a three-dimensional environment for MM cells used microspheres with gel consistency. The microspheres were made of alginate and coated with a polyelectrolyte multilayer produced by the layer-by-layer method. The agglomerate of these microspheres is what we call a microgel. When the cells were grown in the microgel (in the space between the microspheres) the effect was similar to growing them dispersed in a hydrogel. The only difference was that in the microgel, the cells could migrate, and the particles were mobile, permitting the environment to accommodate cell growth or extracellular matrix production [24,25].

In the layer-by-layer procedure [26], the anionic character of the alginate microsphere’s surface was used to electrostatically attach a polycation layer. In this study, type I collagen was attached in an acidic medium. The attraction between the negative charges of the microsphere surface and the positive charges of the polycation allowed the deposition of a polycation layer to coat the microsphere’s surface. A second layer of polyanion could be attached—which, in our case, was hyaluronic acid—producing a second coating, and so on. Multilayer hyaluronic acid coatings and collagen on flat supports have already been studied by Zhao 2014 [27].

Multiple myeloma cells are not adherent in culture; both cells and microspheres are suspended homogenously by orbital shaking in a liquid medium in the desired proportions.

As a proof of concept, we tested cell viability and proliferation with the RPMI 8226 cell line. The study of the RPMI 8226 cell line has particularly attracted the interest of different research groups, and various works in the literature concentrate on the response of this cell line. The goal of this work was to show that microgel is a suitable 3D environment and recapitulates the generation of drug resistance in response to cell interactions with their extracellular matrix components. As a result, the platform application starting off in the MM disease model could extend the study to a broad set of cell lines and biomolecules for drug testing.

To show how our culture platform can be used to test the effect of antitumor treatments, we analyzed the MM cell responses in culture to bortezomib (BRZ) and dexamethasone (DEX). The combination of these two drugs is a commonly used treatment in patients with MM. While BRZ is a proteasome inhibitor, DEX is a corticosteroid [28,29,30], and the synergistic activity of both drugs increases the anti-tumor effect [29].

Proteasome inhibitors prevent the degradation of denatured proteins, which, when accumulated, could cause cell toxicity and induce apoptosis [31]. Bortezomib in MM induces cleavage of survival proteins, such as Mcl1, and triggers apoptosis [31,32], or downregulation, of adhesion molecules such as VLA-4 [33]. On the other hand, DEX induces apoptosis by the activation of intrinsic apoptotic pathways [30,34], a downregulation of antiapoptotic genes, and an upregulation of pro-apoptotic genes [30,35], among other action mechanisms. Studies have been performed on the risk-benefit balance of DEX dosing on patients with MM, where the use of high doses of DEX was found to overcome resistance to the disease despite generating higher toxicity than low-dose delivery [36,37,38].

## 2. Materials and Methods

### 2.1. Microgel Production

**Figure 1:**
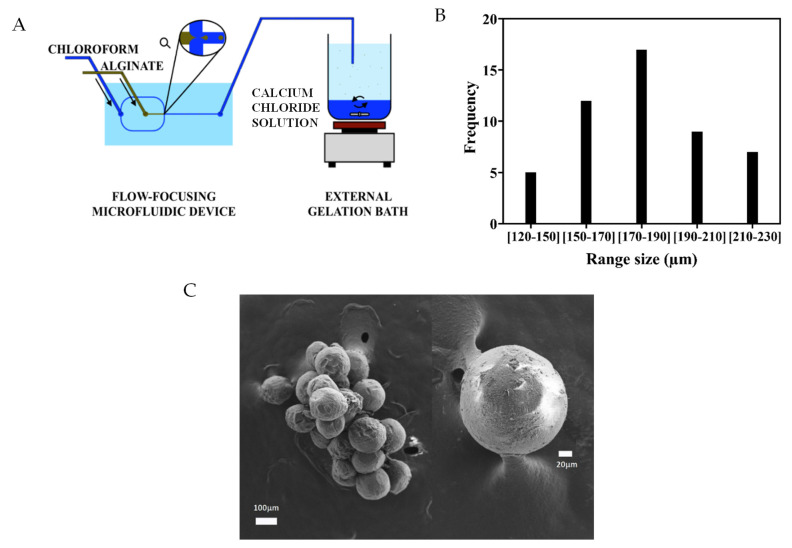
(**A**) Schematic representation of the of the flow-focusing microfluidic device; (**B**) histogram of alginate microspheres diameter; (**C**) FESEM image of a representative microsphere.

The production of alginate microspheres was carried out using a flow-focusing poly (dimethylsiloxane) microfluidic device (Figure 1A inset) with 500 × 500 µm channel size, allowing the production of microspheres with a 170–190 µm average diameter and low size dispersion. For this purpose, a 1.5% *w*/*v* alginate (Sigma-Aldrich, St. Louis, MO, USA) and ultrapure water solution were used as dispersed phase and chloroform (Scharlab, S.L., Barcelona, Spain) as continuous phase; flow rates were set at 0.13 and 6.2 mL/min, respectively. After formation within the device, alginate microspheres were dropped in a gelation aqueous solution of 10% *w*/*v* calcium chloride (Scharlab). Gelation occurs via ionic cross-linking of the alginate chains with calcium ions. After crosslinking, microspheres were collected and thoroughly washed in ultra-pure water.

In order to be visualized by Field Emission Scanning Electron Microscope (FESEM, ULTRA 55 model, ZEISS, Oberkochen, Germany), microspheres were subjected to a fixation protocol—first being immersed in a 3% glutaraldehyde solution (Sigma-Aldrich) in phosphate-buffered saline solution (PBS, Sigma-Aldrich) for 30 min, and then being washed twice in PBS for 10 min. After sample dehydration, they were sequentially immersed in ultra-pure water/ethanol solutions with increasing ethanol content of 30, 50, 70, 80, 90, 95, 100, 100, and 100%, for 10 min each. Finally, samples were dried with supercritic fluid in Leica EM CPD300 equipment (Leica Microsystems Inc., Buffalo Grove, IL, USA), getting them ready to be visualized by FESEM.

### 2.2. Surface Functionalization

Microspheres were functionalized using the layer-by-layer technique, an electrostatic self-assembly method in which alternating layers of collagen (Advanced BioMatrix, Inc., San Diego, CA, USA) and hyaluronic acid (Sigma-Aldrich) are deposited one over the other, as described by Zhao 2014 [27]. Initially, 1 mg/mL collagen (3 mg/mL) and hyaluronic acid (10 g, ~1.5–1.8 × 10^6^ Da) solutions in ultra-pure water were prepared and stirred overnight at 4 °C and pH 5, balanced with HEPES buffer (4-(2-hydroxyethyl)-1-piperazineethanesulfonic acid) (Sigma-Aldrich) to ensure complete dissolution. Microspheres were initially prepared by immersion in ultra-pure water at pH 5 to negatively charge their surface. In the following steps, layer-by-layer depositions were performed in orbital agitation at 600 rpm. First, microspheres were transferred and maintained in the collagen solution for 20 min to deposit the first layer, then washed in ultra-pure water at pH 5 for 20 min to eliminate any non-adhered collagen molecules while keeping collagen coating positively charged. Second, microspheres were transferred and then kept in the hyaluronic acid solution for 20 min to deposit the first hyaluronic acid layer, followed by washing in ultra-pure water for an additional 20 min. The first and second steps were repeated alternatively to produce two sets of microspheres: one with 9 layers of coating (4 layers of hyaluronic acid and 5 layers of collagen), with collagen as the last layer; and another with 10 layers of coating (5 layers of hyaluronic acid and 5 layers of collagen), with hyaluronic acid as the last layer.

### 2.3. Characterization

Micro-BCA assay was performed using a Pierce BCA Protein Assay Kit (Thermo Fisher Scientific, Waltham, MA, USA), applying the standard protocol of the kit to quantify the total amount of collagen after each coating and using microspheres without any coating as baseline alginate. The assay was performed in quadruplicates and read on a Victor3 Plate Reader (PerkinElmer, Waltham, MA, USA). The results were evaluated by averaging the samples at 570 and 550 nm. Moreover, the presence of collagen and hyaluronic acid at the surface was qualitatively assessed by the Fourier Transform Infrared Spectroscopy (FTIR, Bruker Scientific LLC, Billerica, MA, USA) detecting the appearance of their specific absorption peaks.

### 2.4. Cell Culture

Multiple myeloma cell line RPMI 8226, a kind gift from Dra. Beatriz Martin (Josep Carreras Leukaemia Research Institute), were grown in RPMI 1640 (Gibco, Thermo Fisher) and supplemented with 15% fetal bovine serum (FBS, Gibco), 1% L-glutamine (Sigma-Aldrich), and 1% penicillin/streptomycin (Gibco, 10,000 U/mL). 1.5 × 10^5^ cells were cultured in suspension in a 24 well-plate with orbital shaking (VWR, Radnor, PA, USA) at 300 rpm. Conventional culture without agitation was also performed as 2D control. The total volume of the culture medium was 500 µL. In the case of the microgel, the volume ratio microspheres/liquid medium was 20:80 (100 µL dry microspheres/400 µL liquid-medium). The liquid medium was partially renewed every day. To do that, 400 µL of fresh media were added to the culture well, maintaining it in agitation for 15 min to favor the distribution of nutrients. The agitation was interrupted for 1 h to allow cells and microspheres to precipitate to carefully remove 400 µL of the culture medium.

Three replicates per condition were used for flow cytometry studies. Each was analyzed independently in the DEX assay, while in the BRZ and cell cycle assay they were unified into a single tube that was analyzed in the cytometer. In addition, to prevent the equipment obstruction by the alginate microspheres, each sample was passed previously through a 70 µm filter.

### 2.5. Proliferation Assay

The proliferation studies were carried out on days 2, 5, and 7 in two independent experiments. First, cell number was obtained by Quant-iT Picogreen dsDNA kit (Invitrogen, Thermo Fisher). Cell digestion of the samples was performed with a lysis buffer solution (LBS) prepared for 100 mL using 0.653 g of sodium phosphate dibasic (Panreac Quimica SLU, Barcelona, Spain), 0.648 g of sodium phosphate monobasic (Panreac), and 1 mL of EDTA (Gibco, 0.5 M) dissolved in ultra-pure water at pH 6.5. On the day of the digestion, 3.875 U/mL of papain (Sigma-Aldrich) and 1.5 mg/mL of L-cysteine were added to the LBS, and 500 µL were added to each sample and kept under agitation at 60 °C for 18 h. Due to the chelating action of EDTA during the lysis incubation, the alginate of the microspheres becomes soluble and there is no need to separate it from the cells. Once the lysis procedure was completed, DNA was incubated with the PicoGreen solution following the manufacturer’s instructions (4 replicas per condition) to use alginate microspheres without cells as a baseline. Analysis was carried out afterwards on an opaque plate, Optiplate96F (PerkinElmer), using a Victor3 plate reader (PerkinElmer) at 485/535 nm. Secondly, a cell cycle assay was performed by flow cytometry with a DNA-PREP kit (Beckman Coulter, San Diego, CA, USA). Briefly, after two washes with PBS, the samples were centrifuged at 300× *g* for 5 min. Then, 100 µL of DNA PREP LPR permeabilization solution were added to each sample and vigorously vortexed for 10 s, and 1 mL of DNA PREP Stain was added to each sample and incubated in the dark for 30 min at room temperature. DNA PREP Stain contains propidium iodide and RNAse to remove cytoplasmic RNA that can bind with propidium iodide and generate false-positive events [39]. Finally, samples were acquired in a Navios flow cytometer (Beckman Coulter) and data were analyzed on Kaluza 2.1 software (Beckman Coulter). One of the problems with the cell cycle assay by flow cytometry is the formation of cell aggregates that can lead to a generation of doublets of G_0_–G_1_ cells, which can be interpreted as the G_2_-M peak. To avoid this, the acquisition of the events was carried out at a low flow rate, which has the advantage of reducing the formation of aggregates. On the other hand, the cells isolated from the three replicas of each experiment were analyzed in a single flow cytometer tube to have enough events, improving sensitivity and allowing a representative cell cycle analysis.

### 2.6. Viability Assay and CAM-DR Effect

The CAM-DR effect was evaluated by exposing 1.5 × 10^5^ cells to BRZ (STADA, Bad Vilbel, Germany, 2.5 mg/mL) and DEX at 8.75 mg/mL (Fortecortin, Merck KGaA, Darmstadt, Germany) for 48 and 72 h, respectively. In addition, a control without drugs was prepared for each condition (data not shown) to normalize the viability results of each CAM-DR condition. As DEX is used in humans in a broad interval of doses, we performed a pre-test dosage of DEX ranging between 1 and 10^3^ µM in a 2D conventional culture to determine the final concentration of DEX used for the CAM-DR tests.

Cell viability assay was assessed by flow cytometry using Annexin-V kit (Miltenyi Biotec, Bergisch Gladbach, Germany) and 7-Amino-Actinomycin D (7-AAD, Becton Dickinson, San José, CA, USA, EEUU). First, 1.5 × 10^5^ cells were washed two times with PBS and resuspended in 300 µL of 1X binding buffer solution (Miltenyi Biotec). Then, 10 µL of Annexin-V conjugated with fluorescein isothiocyanate (FITC) and 5 µL of CD-138 conjugated with BD Horizon V500 (Becton Dickinson) were added and cells were incubated in the dark for 20 min at room temperature. After an additional wash with 1–2 mL of 1X binding buffer solution, cells were centrifuged at 300× *g* for 5 min and resuspended in 200 µL of 1X binding buffer solution. Then, 10 µL of 7-AAD were added and incubated for 15 min in the dark at room temperature and, finally, the samples were analyzed in a FACSCanto-II flow cytometer (Becton Dickinson) and data analysis was carried out by Kaluza 2.1 software.

### 2.7. Statistical Analysis

The ANOVA one-way analysis (Bonferroni test) was used to determine statistically significant differences between groups by the IBM SPSS Statistics software version 20.0 considering *p* ≤ 0.05 significant, and data were presented as mean ± SD.

## 3. Results

### 3.1. Alginate Microgel

Alginate microspheres were produced with a flow-focusing microfluidic device (Figure 1A). The flow of the dispersed and continuous phases was optimized to obtain microspheres with diameters below 240 microns. Figure 1B,C show a histogram of the microsphere’s diameter and a scanning electron microscopy picture of a microsphere after drying with the critical point method to avoid the collapse of the gel, respectively. The average microsphere diameter was 177 ± 23 μm.

### 3.2. Biomimetic Functionalization

**Figure 2:**
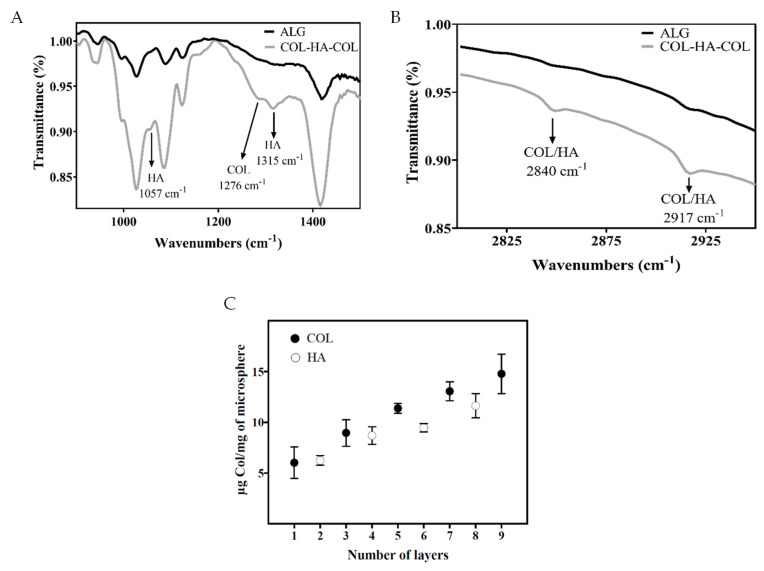
(**A**,**B**) FTIR spectrum of a flat uncoated alginate hydrogel (ALG) and a three-layer layer-by-layer coating collagen-hyaluronic acid-collagen (COL-HA-COL); (**C**) Collagen content in the layer-by-layer coating by adding layers of collagen and hyaluronic acid successively, as determined by Micro-BCA. ALG: alginate hydrogel; COL: collagen; HA: hyaluronic acid.

Layer-by-layer is a suitable technique to coat the surface of the microspheres with a stable layer of a protein, a polysaccharide, or a combination of both. Two series were prepared, one of them with ten layers ending with hyaluronic acid and the other one with nine layers ending with collagen. In order to confirm the coating protocol, an experiment was performed by coating the alginate film’s surfaces and assessing the presence of collagen and hyaluronic acid on the surface by FTIR. The infrared beam is capable of penetrating beyond the thickness of the coating, which is expected to be 30 μm with the implemented coating protocol according to reference [40]. Thus, the spectrum (Figure 2A) reveals the characteristic peaks of the three components of the system: alginate, hyaluronic acid, and collagen. A wet alginate film is also shown for comparison.

Figure 2A shows the characteristic peaks of alginate in the interval of 1200–870 cm^−1^, corresponding to the carbohydrate region, as well as the peak at 1410 cm^−1^, attributed to the symmetric stretching band of the COO-group [41,42]. As for the collagen peaks, the peak at 1232 cm^−1^, corresponding to vibrations on the plane of amide III due to C-N stretching and N-H deformation, stands outwhich, in our case, is slightly above 1276 cm^−1^ and may be due to the interactions of collagen with hyaluronic acid and/or alginate [42,43]. The same situation can be found in Figure 2B with respect to the characteristic peak at 2925 cm^−1^, attributed to the stretching vibration of C-H in both hyaluronic acid and collagen, that in the layer-by-layer surfaces split into one peak at 2840 cm^−1^ and another one at 2917 cm^−1^. This fact can be attributed to interactions between the two components [44,45]. Finally, in the case of hyaluronic acid, Figure 2A shows a peak at 1315 cm^−1^ corresponding to the C-H vibration and a slight increase at 1057 cm^−1^ for C-O stretching and vibration [46].

A quantitative study was carried out to assess the increment of the total amount of collagen in the coating with an increasing number of layers. Micro-BCA analysis (Figure 2C) shows that collagen content increases continuously with the number of collagen- hyaluronic acid bilayers. Following the results shown in Figure 2C, the content of collagen in the first layer adhered to the alginate surface is 6.02 ± 1.55 µg/mg microspheres. Subsequently, after the layer of hyaluronic acid (two layers) was incorporated, the measured value of collagen content was 6.25 ± 0.48 µg/mg microspheres, similar to the one observed in the first layer. This means that the hyaluronic acid layer did not hinder the quantification of collagen present in the first layer. However, differences were observed with the incorporation of the second layer of collagen (three layers) with 8.95 ± 1.29 µg/mg microspheres. With the incorporation of the second layer of hyaluronic acid (four layers), the observed value of collagen content of 8.69 ± 0.85 µg/mg microspheres was slightly lower than that measured in the three layers sample. This behavior is shown in the following bilayers (Figure 2). Finally, with the incorporation of the fifth and last layer of collagen (nine layers), values of 14.77 ± 1.95 µg/mg microspheres were reached. Despite the fact that in the Micro-BCA test, reactants can reach internal collagen layers, hyaluronic acid layers hinder the diffusion in some way through the coating, and the amount of protein is underestimated. Regardless, the continuous increase of the collagen amount shown in Figure 2C proves the deposition of both collagen and hyaluronic acid layers in the coating. A quantitative assessment of the amount of deposited hyaluronic acid was prevented by the chemical similarity with alginate.

### 3.3. Proliferation and Viability

**Figure 3:**
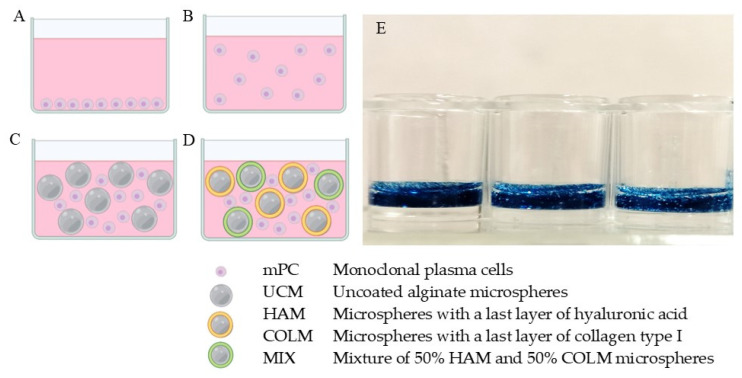
Graphic representation of the different forms of cultivation performed: (**A**) conventional 2D culture; (**B**) suspension culture (SUP condition); (**C**) suspension culture incorporating non-functionalized alginate microspheres (UCM condition); and (**D**) representation of suspension culture in an environment with components of the extracellular matrix incorporating 50% of alginate microspheres with the last layer of hyaluronic acid and 50% of collagen type I (MIX condition). mPC, monoclonal plasma cell; UCM, alginate microspheres; HAM, alginate microspheres with the last layer of hyaluronic acid; COLM, alginate microspheres with the last layer of collagen type I. (**E**) shows a picture of alginate microspheres stained with Alcian blue and suspended in the aqueous medium of the same proportion as the cell culture.

Six series of culture conditions were considered: conventional 2D control culture (2D), suspension control (SUP), control with uncoated alginate microspheres (UCM), microspheres with the last layer of hyaluronic acid (HAM), microspheres with the last layer of collagen (COLM), and as the final condition, a mixture of 50% HAM and 50% COLM microspheres (MIX) was used. A schema of the culture series is represented in Figure 3. In all the systems, mPC cells were cultured in suspension in the liquid medium, instead of a conventional 2D culture. In the case of the microgels, in addition to the liquid medium, the environment of the cells contained the microspheres that exhibit the biomolecules of interest in this study. To show the homogeneity of the suspension of the microspheres during culture, alginate microspheres were stained with Alcian blue and dispersed in the aqueous medium in the same proportion as in the cell cultures. Figure 3E shows a picture of the suspension on the orbital shaker. Following the culture, microspheres were extracted by filtration and suspended cells were analyzed with the different techniques. The fact that cells were cultured in suspension differentiates the microgel from a 3D scaffolding system in which cells and material would be closely packed or formed by cells encapsulated in a gel; in our case, microspheres are not porous, as seen in Figure 1C, so cells cannot penetrate the microspheres.

The influence of the presence of hyaluronic acid and collagen in the microgel on cell proliferation was assessed by the cell cycle test and the cell number measured as total DNA content in the Picogreen test on days 2, 5, and 7. On the other hand, a study of cell viability was performed to corroborate the non-cytotoxicity of the materials used. In general, the results showed significant differences among the various systems on days 2 and 5, while on day 7, the culture became saturated as the volume of the well and the supply of nutrients became insufficient for the attained number of cells. As a consequence, all the systems presented similar results.

**Figure 4:**
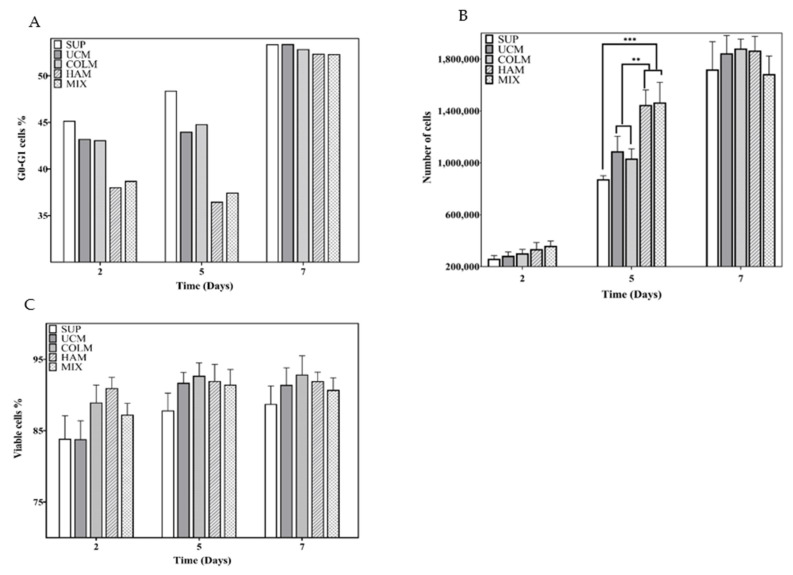
(**A**) Fraction of cells in G_0_–G_1_ phases; (**B**) cell number in the different culture environments; (**C**) percentage of cell viability. The level of statistical significance is shown as: (**) *p*-value ≤ 0.01; (***) *p*-value ≤ 0.001.

The cell cycle of cells cultured in UCM and COLM conditions showed a small and remarkable decrease in the fraction of cells arrested in the G_0_–G_1_ phases with respect to the SUP condition on days 2 and 5 (Figure 4A) which, at the same time, was responsible for the slight increase in cell number observed on day 5 (Figure 4B). This increase was approximately 2.15 × 10^5^ ± 1.16 × 10^5^ and 1.56 × 10^5^ ± 7.4 × 10^4^ cells for UCM and COLM, respectively, as compared to SUP. Consequently, as the results with COLM were very similar to the UCM results, we can deduce that the presence of collagen does not alter the proliferative capacity of the mPCs. However, the presence of microgels when the last layer is hyaluronic acid yields the improvement of cell proliferation. In the cell cycle assay, a clear decrease in the fraction of cells arrested in the G_0_–G_1_ phases between the HAM and MIX conditions was observed with respect to SUP, UCM, and COLM (Figure 4A). This effect was corroborated by the PicoGreen results, where there was a slight increase in cell number on day 2, but a significant difference on day 5 of culture. The cell number at day 5 in the HAM and MIX conditions was 5.73 × 10^5^ ± 1.2 × 10^5^ and 5.92 × 10^5^ ± 1.55 × 10^5^ cells higher than in the SUP condition, respectively. The difference with respect to UCM and COLM was smaller but still significant: 3.6 × 10^5^ and 3.76 × 10^5^ cells higher than in the UCM condition, respectively, and 4.13 × 10^5^ and 4.32 × 10^5^ cells higher than in the COLM condition, respectively (Figure 3B). In this way, the use of both techniques allowed us to corroborate that the presence of hyaluronic acid promotes cell proliferation while the presence of collagen does not.

On day 7 of the culture, it was possible to see how the cell number in each condition was very similar (Figure 4B). This clearly indicated that the platform had reached its saturation level due to the high growth rate of this cell line. The viability results (Figure 4C) showed that even when the culture was saturated, there was no increase in cell mortality, as each condition maintained an average viability of 91 ± 2.3% at day 7. These data are relevant since, as shown in Figure 4B, there was a considerable reduction in the cell number between days 5 and 7 in each of the conditions, more remarkable in the HAM and MIX conditions. Therefore, the idea that this reduction in proliferation was a result of the presence of dead cells can be discarded. The cell cycle assay presented an increase in the percentage of cells arrested in G_0_–G_1_ phases in each of the conditions on day 7 compared to day 5, with differences of 4.98% in the SUP condition, 9.41% in UCM, and 8.05% in COLM. This increase was relevant in the HAM and MIX conditions (15.88% and 14.86%, respectively). The increase in the percentage of cells in the G_0_–G_1_ phase due to culture saturation meant that the cells were maintained in a quiescent state, explaining the decrease in the number of cells between days 5 and 7 observed in Figure 4B.

### 3.4. Drug Resistance

**Figure 5:**
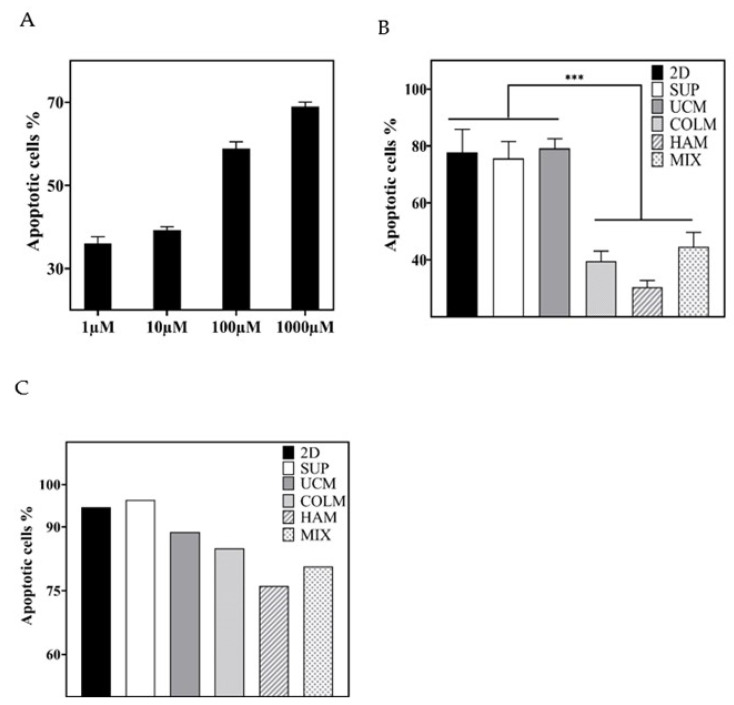
Percentage of apoptotic cells after exposition to DEX (**A**,**B**) and BRZ (**C**). 2D culture at varying DEX dose (**A**); influence of the presence of hyaluronic acid or collagen on the development of drug resistance (**B**,**C**). The level of statistical significance is shown as (***) *p*-value ≤ 0.001. 2D, conventional 2D control culture; SUP, suspension control; UCM, control with uncoated alginate microspheres; HAM, microspheres with a last layer of hyaluronic acid; COLM, microspheres with a last layer of collagen; MIX, mixture of 50% HAM and 50% COLM microspheres.

Apoptotic cells were evaluated by flow cytometry through Annexin-V and the 7-AAD staining method. Due to the clinical repercussions of using a low or high dose of DEX in MM patients [36,37,38], a viability test was performed to adjust the DEX dosage. This experiment was carried out in a conventional 2D culture at 72 h, varying the dosage from 1 to 1000 µM. The results shown in Figure 5A support the use of a 1000 µM concentration in CAM-DR trials, which produce mortality of around 70%. After 72 h of DEX exposure under SUP control conditions, no significant differences were observed among the conventional 2D culture (77.78 ± 8.1%), SUP (75.64 ± 5.95%), and UCM (79.17 ± 3.35%) (Figure 5B). However, when the microgel was functionalized, the mortality decreased, reaching values of 30.40 ± 2.41% for the HAM, 39.51 ± 3.63% for COLM, and 44,56 ± 5.11% for the MIX, which meant a reduction of 39% with respect to control conditions (Figure 5B).

In the case of BRZ, since no relevant literature was found on the impact of the use of various treatments using low or high doses of BRZ on MM patients, we decided to establish a known concentration found in the literature (10 nM) for the assay [47]. Therefore, after 48 h of drug exposure, high mortality was observed in control conditions, reaching 94.64% in 2D, 96.37% in SUP, and 88.77% in UCM. In the HAM and COLM microgels, the mortality was also quite high but lower than in the control conditions: 76.2% in HAM, 84.9% in COLM, and 80.7% in MIX (Figure 5C). Due to the high mortality presented 48 h after cell culture, the three replicates of the BRZ samples were unified into a single flow cytometry tube to provide sufficient events for the results to be representative.

## 4. Discussion

### 4.1. Biomimetic Microgel as a 3D Culture Environment for mPCs

The development of a realistic in vitro disease model for MM that could be used to discover new antitumoral drugs or to personalize patient treatments is still a challenge. Such a culture platform requires an artificial three-dimensional environment in which the mPCs generate the same resistance to drugs as they do in vivo. The problem represented a huge challenge due to the importance of the medullar niche in disease development, where the adhesion of myeloma cells to bone marrow stromal cells and extracellular matrix has been shown to influence proliferation, growth, invasion, and drug resistance of mPCs [18]. In a microenvironment lacking the key biomolecules that interact with mPCs, such as conventional 2D culture, the cellular response to the drug is altered due to its unnatural niche [36]. In addition, there is evidence that the behavior of the mPCs in a 3D culture differs morphologically and physiologically from cells in 2D culture [37]. Due to this issue, the development of new 3D systems that mimic the in vivo cell behavior and provide more predictable results to in vivo tests is being performed [16,17].

The aim of this study was to carry out the development of a culture platform based on a microgel and to corroborate that the system allows an effective interaction of the mPC with the extracellular matrix components on the surface of the microspheres. Although MM is a heterogeneous disease, the study was performed on the RPMI 8226 cell line, one of the most studied cell lines in MM, as evidenced by the large number of literature on the subject. In vitro tests with this cell line allowed us to confirm that the response observed in our system was the result of the interaction between mPC and the biomolecules included in the surface of the microgel. These results lay the foundation of a dynamic 3D culture model that might, in the future, be used for drug assays in the primary cells of MM patients. Microgels constitute an environment closer to the in vivo niche than the conventional 2D culture. In this way, the implication of two biomolecules, hyaluronic acid and collagen, have been proven on mPCs proliferation and CAM-DR effect towards DEX and BRZ. In comparison to other 3D platforms, where cells are confined or aggregated in the form of spheroids, the system developed by maintaining the culture in suspension at a 20:80 microgel/cell volume ratio allows larger cell mobility in comparison to hydrogels. In addition, microgel configuration permits the maintenance or even the increase of cell-cell contact, while favoring the interaction of mPCs with the biomolecules present in the microgel.

The microsphere functionalization method selected was layer-by-layer, a fast and simple coating technique. At the same time, it provided a great versatility to the developed platform to simultaneously perform the study of different biomolecules well. The stability of the collagen/hyaluronic acid’s layer-by-layer coating in the culture medium has been proven by Zhao 2014 [27], who maintained flat substrates with this coating for up to 14 days in culture. On the other hand, the stability of the alginate core of the functionalized microspheres was assessed in the assays performed using uncoated alginate microspheres in up to 5-day-old cultures in proliferation and drug resistance tests. Moreover, in polyelectrolyte multilayers, bonding between layers is based on electrostatic interaction; this opens a wide range of possibilities to design and manufacture in mild conditions microgels functionalized with different biomolecules. Thus, heparin, heparan sulphate, chondroitin sulphate, or other collagens hypothesized to be involved in CAM-DR could be incorporated into the 3D environment with the procedures discussed here. Even formulation including microspheres functionalized with different biomolecules can be designed to mimetize the bone marrow niche, as we did in this work with the MIX microgel. Such an environment can be used to further study the individual or synergic effects of bone marrow extracellular matrix components on CAM-DR against different known or new drugs, or even to develop drugs capable of inhibiting the interaction of mPCs with specific biomolecules.

In this line, as a proof of concept, the effect of hyaluronic acid and collagen on mPC cells has been studied. The results obtained in this work show that, although there are no significant differences in terms of cell proliferation between SUP, UCM, and COLM conditions, a slight increase in the number of cells in the UCM and COLM conditions with respect to SUP can be appreciated (Figure 4B). This may be due to the presence of our developed 3D culture system based on microgel, which allows increased cell-cell contact (Figure 3C,D), slightly favoring cell proliferation. However, when the last layer of the coating is hyaluronic acid, cell proliferation significantly increased on day 5 with respect to the rest of the conditions (Figure 4B). The same proliferative behavior was observed in the MIX condition, where the proliferative effect was attributed to the presence of HAM microspheres, since COLM did not show any influence on proliferation. Even if the coating contains both biomolecules, the cellular response when the last layer is hyaluronic acid or collagen is completely different. It is known that hyaluronic acid in human IL-6-dependent cell lines induced proliferation through an IL-6-mediated pathway involving the phosphorylation of retinoblastoma proteins [10]. This suggests that the mechanism of action of hyaluronic acid on the proliferation of RPMI 8226 IL-6-independent cells acts through signaling pathways different than in the IL-6-dependent MM cell line. This fact is not surprising since, with the same cell line, it was observed that the drug resistance induced by hyaluronic acid to DEX was mediated through distinct signaling pathways in IL-6-independent and IL-6-dependent MM cell lines [11].

### 4.2. RPMI 8226 Cell Line in the Presence of Hyaluronic Acid or Collagen Generates Drug Resistance to Dexamethasone

The implication of hyaluronic acid in drug resistance in the cell line RPMI 8226 has already been described in 2D cultures [11]. In our 3D culture platform, the interaction with HAM microspheres reduced mPC mortality significantly, to around 39%, when compared with control conditions (Figure 5B). The same reduction was observed in collagen conditions, although the resistance of the RPMI 8226 cell line to DEX induced by collagen has not been previously described. Further research will be necessary to clarify the mechanisms of collagen-induced drug resistance on MM cells. As the union to collagen is mediated by syndecan-1, which, in turn, promotes Wnt signaling, our strongest hypothesis is that it may be responsible for the drug resistance [12,15].

In the case of BRZ, cell viability in UCM and in the microgels functionalized with hyaluronic acid or collagen in the last layer was higher than in 2D and SUP conditions (Figure 5C). This difference may be the result of the favored cell-to-cell contact in a microgel environment which, in turn, slightly favors cell proliferation, resulting in the presence of a higher percentage of viable cells, as mentioned above. However, these differences are not significant enough to attribute to hyaluronic acid and collagen a CAM-DR effect against BRZ, as occurs with DEX.

Therefore, with the generation of these results, our work proves the utility of the culture platform in testing the effect of drugs targeting the interaction of mPCs with specific biomolecules or ligands by functionalizing the microspheres with those biomolecules and performing viability studies. This represents a step further in the development of realistic disease models for MM. Once the interaction of the microgel with the MM cell line has been confirmed, the validation of the culture system using primary cells from MM patients appears as a short-term future perspective.

## 5. Conclusions

We presented a culture platform based on a biomimetic microgel formed by alginate microspheres whose surface has been coated with certain biomolecules of the bone marrow extracellular matrix. This represented a three-dimensional system, where mPCs were grown in suspension in a liquid medium but separated by distances similar to the cell sizes themselves, as the bone marrow components were capable of generating drug resistance. As a proof of concept, the effect of hyaluronic acid on the proliferation and generation of DEX resistance in the RPMI 8226 cell line has been proved. We also showed how the presence of collagen did not have a significant influence on proliferation and yet was capable of inducing resistance to DEX. The behavior of a microgel with half of the microspheres with hyaluronic acid on their surface, and collagen on the other half, was analogous to a microgel with all the microspheres coated with hyaluronic acid. The lack of CAM-DR effect against BRZ, induced by the interaction with hyaluronic acid and collagen, has been confirmed. Although our results bear the limitation of having been obtained with a single cell line, this work shows a promising view in terms of establishing microgels functionalized with components of the bone marrow extracellular matrix as a disease model for MM. Additional analysis of other cell lines and biomolecules will be necessary to extend the use of this culture platform for studies involving patient cells.

## Data Availability

Publicly available datasets were analyzed in this study. This data can be found here: Riunet.upv.es.
